# Comparative Outcomes and Surgical Timing for Operative Fragility Hip Fracture Patients during the COVID-19 Pandemic: A Retrospective Cohort Study

**DOI:** 10.3390/geriatrics7040084

**Published:** 2022-08-13

**Authors:** Katherine A. Rowe, Kiryung Kim, Nathan H. Varady, Marilyn Heng, Arvind G. von Keudell, Michael J. Weaver, Ayesha Abdeen, Edward K. Rodriguez, Antonia F. Chen

**Affiliations:** 1 School of Medicine, Harvard Medical School, Harvard University, Boston, MA 02115, USA; 2 Department of Orthopaedic Surgery, Hospital for Special Surgery, New York, NY 10021, USA; 3 Department of Orthopaedic Surgery, Massachusetts General Hospital, Boston, MA 02114, USA; 4 Department of Orthopaedic Surgery, Brigham and Women’s Hospital, Boston, MA 02115, USA; 5 Bisbebjerg Hospital, Department of Orthopaedic Surgery, Division of Orthopaedic Trauma, University of Copenhagen, 2400 Copenhagen, Denmark; 6 Carl J. Shapiro Department of Orthopaedics, Beth Israel Deaconess Medical Center, Boston, MA 02215, USA

**Keywords:** hip fracture, COVID-19, COVID-19 testing, time-to-surgery

## Abstract

The COVID-19 pandemic had wide-reaching effects on healthcare delivery, including care for hip fractures, a common injury among older adults. This study characterized factors related to surgical timing and outcomes, length-of-stay, and discharge disposition among patients treated for operative hip fractures during the first wave of the COVID-19 pandemic, compared to historical controls. A retrospective, observational cohort study was conducted from 16 March–20 May 2020 with a consecutive series of 64 operative fragility hip fracture patients at three tertiary academic medical centers. Historical controls were matched based on sex, surgical procedure, age, and comorbidities. Primary outcomes included 30-day mortality and time-to-surgery. Secondary outcomes included 30-day postoperative complications, length-of-stay, discharge disposition, and time to obtain a COVID-19 test result. There was no difference in 30-day mortality, complication rates, length-of-stay, anesthesia type, or time-to-surgery, despite a mean time to obtain a final preoperative COVID-19 test result of 17.6 h in the study group. Notably, 23.8% of patients were discharged to home during the COVID-19 pandemic, compared to 4.8% among controls (*p* = 0.003). On average, patients received surgical care within 48 h of arrival during the COVID-19 pandemic. More patients were discharged to home rather than a facility with no change in complications, suggesting an opportunity for increased discharge to home.

## 1. Introduction

Fragility hip fractures are a common injury among older adults, and the COVID-19 pandemic has affected many aspects of surgical care for patients with such fractures. While the COVID-19 pandemic may have decreased the number of patients seeking care for other fragility fractures, such as the forearm, humerus, and ankle, it did not decrease the number of patients requiring inpatient hospitalization for hip fracture care [1]. Additionally, while other types of emergency surgery may have decreased during the pandemic, hip fracture admissions remained constant or increased [2,3,4,5,6,7,8]. COVID-19 infection is an established risk factor for mortality among hip fracture patients in comparison to COVID-19 negative patients. Numerous studies across geographies have suggested that hip fracture patients who tested positive for COVID-19 had increased mortality [9,10,11,12,13,14,15,16,17]. Furthermore, meta-analyses have found a four- to seven-fold increase in mortality among patients with COVID-19 infection in comparison with those treated during the COVID-19 pandemic who did not have COVID-19 [18,19,20,21,22].

Given the pandemic’s widespread impact on all aspects of healthcare delivery, it is also essential to understand outcomes for patients with hip fractures regardless of COVID-19 status during this period. The management of fragility fractures requires expedient interdisciplinary care to minimize morbidity and mortality [23]. During the COVID-19 pandemic, shifts in resource needs and disruption to normal health system functioning may have impaired the ability to provide this care across geographies [24,25]. This has prompted calls to reprioritize providing comprehensive fracture care to prevent significant societal costs [26]. Epidemiologic studies have suggested that patients who sustained hip fractures during the COVID-19 pandemic might also have increased frailty and decreased mobility at baseline, compared to those who sustained hip fractures prior to the pandemic [27]. Outside of the United States, studies including a large meta-analysis found no difference in hip fracture patient mortality during the COVID-19 pandemic, compared to historical controls [28,29]. Within the United States, an investigation by Egol et al. [30] found an increase in mortality and major complications among hip fracture patients treated during the pandemic, including systemic infections (e.g., urinary, pneumonia, sepsis); surgical site infection; venous thromboembolism; myocardial infarction; stroke; and pulmonary complications. A study in Colorado also found an increase in in-hospital mortality [31]. Studies in other United States geographies that compared hip fracture treatment during the pandemic to historical controls are lacking, although a country-wide claims database analysis suggested no change in mortality or complications [32]. Investigating regional patterns may reveal differences that reflect the various sociopolitical and healthcare systems responses to the pandemic. The role of the COVID-19 pandemic and its associated changes to the healthcare system have not yet been investigated in patients in the New England region of the United States.

A key component of the perioperative period that may impact outcomes is time-to-surgery. Time-to-surgery in hip fracture surgery during the pandemic varied between studies, with select studies reporting a decrease [3,33], others reporting an increase [28,34], and others reporting no change [30,31,35,36,37] in time-to-surgery, compared to historical controls. Furthermore, there are numerous factors that impact overall time-to-surgery. Thus far, investigations into perioperative timing during the COVID-19 pandemic have been limited to time from injury to arrival [31], and timing of anesthesia and holding or transport times [38]. These factors are important to understand, particularly given the impact that anesthesia type may have on surgical timing. No studies to date have investigated the time required for COVID-19 testing alongside time-to-surgery. Thus, this study aims to evaluate the time-to-surgery and associated factors, including time to obtain a COVID-19 test result and anesthesia type, alongside discharge disposition, length-of-stay, mortality, and post-operative complications for operative hip fracture patients. Specifically, the study investigates these factors during the first wave of the COVID-19 pandemic at three academic medical centers in the New England region of the United States and compares these patients to historical controls. It outlines the potential impact of preoperative testing and changes to the operations of the healthcare system on the aforementioned variables, whether patients had COVID-19 or not.

## 2. Materials and Methods

A retrospective, observational cohort study with a historical matched cohort was conducted that included 64 consecutive operative hip fracture patients treated during the first wave of the COVID-19 pandemic. The patients were treated at three academic, tertiary hospitals in the New England area (United States) between 16 March 2020 and 20 May 2020. Institutional Review Board (IRB) approval was obtained for this study at each site for a retrospective exempt study. Thus, informed consent was not required by the IRB.

The included patients underwent cephalomedullary nailing, open reduction and internal fixation, total hip arthroplasty, hemiarthroplasty, or percutaneous pinning for the repair of an operative hip fracture during the study period. A total of 101 patients were initially identified based upon these criteria, of which 10 were excluded because they did not present with operative hip fractures. Patients with high-velocity trauma (12 patients); periprosthetic fractures (4 patients); pathologic fractures (7 patients); and insufficient data to assess outcomes at 30 days (4 patients) were excluded. The included patients had AOTrauma/Orthopedic Trauma Association (AO/OTA) type 31A or 31B fractures [39]. All remaining 64 patients were analyzed. Historical controls were selected from 15 March 2018 to 15 March 2020. The controls were identified using current procedural terminology (CPT) codes 27248, 27245, 27244, 27235, 27236, 27125 + s72.*, and 27130 + s72.*. Exact matching (1:1) was performed for sex, American Society of Anesthesiology (ASA) score, hospital location, and surgical procedure, with propensity matching for age to control for possible confounders.

Sociodemographic information; baseline comorbidities and presentation; the presence of a do-not-resuscitate (DNR) or do-not-intubate (DNI) order at admission; laboratory values and exam findings at admission; COVID-19 testing status; fracture and surgery characteristics; and postoperative outcomes were collected via chart review. Comorbidities collected included those in the Charlson Comorbidity Index [40] (CCI), hypertension, obesity, asthma, and osteoporosis. Basic laboratory values including complete blood counts were gathered, and respiratory findings on exam or chest imaging if performed were noted. The time in hours from emergency department triage to the result of a final COVID-19 test and to surgical procedure start were calculated. Thirty-day complications of interest included mortality, readmission, reoperation, surgical site infection, pneumonia, gastrointestinal complications (emesis), venous thromboembolism, myocardial infarction, stroke, sepsis, or anemia or bleeding requiring the escalation of care beyond transfusion. Additional data on weight-bearing status at discharge, discharge disposition, and length of stay were also collected. A subgroup analysis was conducted to compare patients who received a COVID-19 test to their historical controls. Patients with missing data were excluded from the analysis, given that the majority of patients had data for all key variables of interest. The number of analyzed participants was indicated in parentheses, where patients with missing data were excluded.

Demographic variables, time-to-surgery, and postoperative complications were compared. Continuous variables were compared using two-tailed homoscedastic t-tests, and categorical variables were compared using Chi-squared tests. All quantitative variables were treated as continuous variables. A subgroup analysis was conducted to compare only patients who received preoperative COVID-19 tests and their matched historical controls. Statistical significance was defined as *p* < 0.05. The statistical analysis was performed using SAS v9.4 (SAS Institute, Cary, North Carolina, United States) and Microsoft Excel for Mac Version 16.43 (Redmond, Washington, DC, USA).

A total of 64 study group patients and 64 controls were included in the analysis. There were no differences in baseline characteristics between the groups (Table 1).

## 3. Results

### 3.1. Baseline Characteristics

Demographic variables between the study group patients treated during the COVID-19 pandemic and the controls did not differ (Table 1). There were no differences in average ASA, average CCI, or comorbidities between groups (Table 2).

### 3.2. COVID-19 Testing and Time-to-Surgery

**Figure 1:**
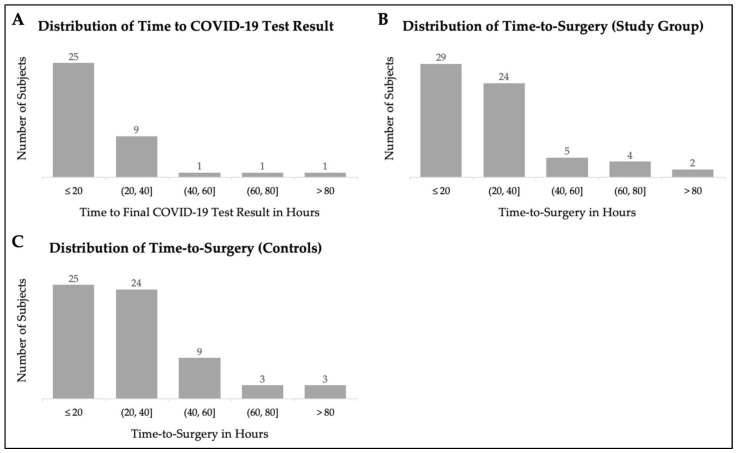
Distribution in time to COVID-19 test result (**A**) and time-to-surgery for study group (**B**) and control subjects (**C**).

Among the patients admitted during the COVID-19 pandemic, 37 patients (58%) received COVID-19 polymerase chain reaction (PCR) testing. Twelve patients (32% of tested patients) received more than one test. Two patients (5% of tested patients) tested positive for COVID-19. Of the two patients who tested positive for COVID-19 on admission, both were symptomatic, and one had multiple complications including congestive heart failure exacerbation and sepsis, while the other had no post-operative complications. Both of these patients underwent surgery within 48 h of presentation. The mean time from arrival to the last COVID-19 test result before surgery was 17.6 h (Figure 1A). Ten patients had chest imaging (X-ray or computed tomography scan) that was concerning for consolidation and possible pneumonia. Six patients required supplemental oxygen on admission in the study group, compared to seven patients among controls (*p* = 0.77). The mean time from arrival to surgery (Figure 1B,C) was 28.0 h for the study group and 28.6 h for controls, with no difference between groups (*p* = 0.88).

### 3.3. Surgical and Anesthesia Characteristics

The patients were matched by surgery type (Table 1). Anesthesia type was similar between groups (*p* = 0.13), with spinal/local anesthesia administered for 19% of study group participants and 9% of controls. There were no conversions from spinal/local to general anesthesia in our study population.

### 3.4. Outcomes, Length-of-Stay, and Discharge Disposition

The mean length of stay was 6.4 days in the study group (range 0–46 days) and 6.0 days among controls (range 1–20 days), and these did not differ between groups (*p* = 0.72). There were three deaths in the study group patients and four deaths in the control patients at 30-days (*p* = 0.70). A detailed analysis of individual complications was conducted, and there were no other differences in individual 30-day complication rates between groups (Table 3). Of the patients who were discharged, more patients were discharged home with services than to another facility during the COVID-19 pandemic—15 of 63 (23.8%) study group patients were discharged home, and 3 of 62 (4.8%) control patients were discharged home (*p* = 0.003). The patients who were discharged home received additional services, such as physical therapy or home healthcare. Three patients in the study group were readmitted for known COVID-19 infection. All of these patients were discharged to rehabilitation facilities and were readmitted within 30 days, although time-to-readmission varied.

### 3.5. Subgroup Analysis

A subgroup analysis was conducted only comparing patients who received a COVID-19 test (*n* = 37) with their historical controls. Within this subgroup, the mean age was 79.6 years ± 11.0 in the study group and 79.5 years ± 10.7 in controls, and there were 22 females (59%) in each group. There was no difference in BMI, race or ethnicity, prevalence of DNR/DNI orders, or fracture type between subgroups. The Charlson Comorbidity Index was 6.8 ± 3.0 in the study group and 6.6 ± 2.5 in controls, and the mean ASA was 3 in both groups (range 2–4). There was also no difference between white blood cell count (*p* = 0.71) or neutrophil-lymphocyte ratio (*p* = 0.92) on admission (*n* = 35 for the study group, *n* = 36 for controls) for these patients who underwent COVID-19 testing versus controls. The mean time-to-surgery was 34.5 h for the study group and 31.4 h for controls (*p* = 0.59). There was no difference in the type of anesthesia delivered (*p* = 0.33). Outcomes, including length of stay (*p* = 0.43); mortality (*p* = 1.00); and 30-day complication rates (*p* = 0.35) also did not differ between groups. Subgroup patients treated during the COVID-19 pandemic were again more likely to be discharged home than to another facility than the historical controls (*p* = 0.006).

## 4. Discussion

Given the significant differences in geographic impacts of and responses to the COVID-19 pandemic, our study sought to investigate the associated impact of the pandemic on hip fracture care for patients at three tertiary academic medical centers in New England in the United States. In particular, we investigated the pandemic’s potential effect on time-to-surgery, anesthesia type, and discharge disposition, and their possible correlation with postoperative outcomes.

Our retrospective analysis comparing 64 patients treated for operative hip fractures during the first COVID-19 wave and historical controls matched on age, sex, surgical procedure, and ASA score did not reveal any differences in the rates of 30-day complications or other adverse surgical outcomes between groups. Our study builds upon what is already known about mortality in hip fracture patients during the COVID-19 pandemic. Much of the early investigation compared patients who tested positive for COVID-19 and those who did not. Studies in Spain [9,15], the United Kingdom [10,11,12,14,16,17], and the United States [13] have suggested increased mortality in hip fracture patients who tested positive for COVID-19 in comparison to those who did not. Additional factors linked to mortality during the pandemic have included male sex; increased age; surgery type (i.e., cephalomedullary nailing); smoking; comorbidity burden; and non-operative management [10,12,15]. These findings of increased mortality in hip fracture patients with COVID-19 compared to those without have been replicated in meta-analyses [18,19,20,22]. Importantly, however, these studies did not utilize historical controls and instead compared patients treated during the pandemic to one another.

Studies of patients regardless of COVID-19 status have yielded conflicting results when comparing the mortality of hip fracture patients treated during the COVID-19 pandemic to historical controls. Increased mortality was found in a cohort of 43 hip fracture patients in the United Kingdom compared to the same time period during the three years prior [41], and a meta-analysis of 2651 patients found an excess mortality of ~10% during the pandemic [21]. Another study in Spain, however, did not find a difference in 30-day mortality, similar to our study [28]. Additionally, a meta-analysis of 1586 hip fracture patients found no difference in 30-day mortality for patients treated during the COVID-19 pandemic versus historical controls [29]. Our findings align with these studies. Differences in the regional impact of and responses to the pandemic also likely impact these findings. Studies in the United States have been limited thus far. Initial small studies suggest increased mortality; a series of 138 hip fracture patients in New York found a 12.3% mortality rate, compared to a 3% 30-day mortality rate in historical controls [30], and a study in Colorado of 351 cases and 352 controls found an in-hospital mortality rate of 3.4% during the pandemic, increased from 1.1% prior [31]. In a national claims database study, in-hospital mortality did not differ between pandemic-era patients and historical controls, although importantly, this study did not capture regional variation [32]. Of note, the study by Egol et al. [30] also found an increase in major complications during the COVID-19 pandemic. This contrasts with our study, where there were no differences in either mortality or complication rates between hip fracture patients treated during the pandemic and historical controls. Importantly, our study was limited by the number of treated patients during the first wave of the pandemic and, therefore, was likely underpowered to detect such a difference in mortality. It is also possible that differences in the magnitude of and response to the first wave of the COVID-19 pandemic between different geographic locations in the United States may have affected outcomes. While our region experienced a meaningful case load, these findings are not generalizable to healthcare systems that experienced greater upheaval due to patient volumes and high death rates during the pandemic. Most of the core functions of our institution persisted despite disruptions. Similar findings were replicated in our study when performing the subgroup analysis of only patients who received COVID-19 testing, compared to historical controls.

In a recent qualitative study, hip fracture care providers reported delays in care including increased anesthesia time, increased time-to-surgery due to COVID-19 testing, and decreased efficiency due to understaffing during the pandemic [42]. Our study found the opposite, as there was no difference in time-to-surgery between patients treated during the pandemic and historical controls, despite a mean of 17.6 h between arrival and the final COVID-19 PCR test result prior to surgery. Additional preoperative workup was concurrent with COVID-19 testing. Importantly, given the ideal to treat operative hip fractures within 48 h to avoid adverse outcomes [23], it did not appear that testing for COVID-19 delayed surgery during the first wave compared to historical controls. Of note, this study was performed at three large, tertiary academic medical centers that developed significant capabilities for in-house COVID-19 testing, resulting in the majority of these tests eventually being performed in a timely fashion. Similarly, it is likely that the nuances of operating in a pandemic necessitated additional cleaning, planning, and preparation prior to surgery. It is not apparent that these processes impacted time-to-surgery negatively. This aligns with the majority of studies conducted to date, which suggest that time-to-surgery for hip fracture patients has not changed during the COVID-19 pandemic [30,31,35,36,37]. Changes to timing of other aspects of the healthcare system have been posited to impact outcomes, such as changes in time to orthogeriatric or multidisciplinary care [37,43]. Arafa et al. [38] found an increase in anesthesia preparation and induction time, although this was only significant in patients who were positive for COVID-19 compared to historical controls. Further investigations into the timeline of perioperative events may reveal additional factors that might influence overall time-to-surgery. Importantly, elective cases were intermittently on hold at our institutions throughout this study period, which is likely to have improved surgeons’ ability to schedule urgent cases. This effect was somewhat counterbalanced, however, by the conversion of numerous operating rooms into areas for intensive care unit-level care for patients who contracted COVID-19.

During the COVID-19 pandemic, there was an increase in the number of patients who were discharged to home as opposed to rehabilitation facilities or other hospitals. This may indicate a desire to transfer patients out of healthcare facilities to home, possibly decreasing the risk of contracting COVID-19 within the hospital or outside facilities. Outbreaks of COVID-19 at select skilled nursing facilities in our catchment area may have affected public perception of the safety of these facilities and decrease patient/patient family desire to be discharged to them. This contrasts with the findings of Wright et al. [43], where 47% of hip fracture patients required increased support or a higher level of care at discharge. A similar study in the United Kingdom found patients were less likely to return home after hip fracture surgery during the pandemic [44]. Many of the patients in our patient population were discharged home with services instead of going to rehabilitation or skilled nursing facilities. Similar findings of increased discharge to home have been replicated in Israel [45], New York City [46], and in a national claims database study in the United States [32] during the pandemic. Notably, this suggests that more patients may be suitable for discharge directly to home than anticipated prior to the pandemic. This may represent a future opportunity for changes to discharge disposition practices. During the time of the study, there were shifts in the availability of home care that may have affected this dynamic. The availability of professional home services may have been stretched given increased demand, as patients were less able to go to healthcare facilities for their care, but families may have been more able to provide home care themselves if they were working from home. The increased ability of family to provide assistance may be a persistent shift as work-from-home options become more prevalent in the wake of the pandemic.

The lack of a difference in the use of spinal or local rather than general anesthesia suggests that anesthesiologists may have remained willing to perform aerosol-generating procedures such as intubation during the pandemic. Studies have reported varying increases in the use of regional anesthesia during the pandemic. A study of 76 hip fracture patients and controls in the United Kingdom found an increase in the use of regional anesthesia during the COVID-19 pandemic from 33% to 63%, but noted that 14% of cases required conversion to general anesthesia [36]. Such conversions did not occur in our population. Another meta-analysis of 1586 hip fracture patients treated during the COVID-19 pandemic in comparison to historical controls found a decrease in general anesthesia from 44.86% to 33.71% [29]. Our study was adequately powered to detect a difference with the magnitude of the former study, but not the latter. We can, therefore, only exclude a large (~25%) increase in local anesthesia utilization.

The limitations of this study included small sample size, limited generalizability given academic medical center sites, and limited follow-up to 30 days postoperatively. While all eligible patients with operative hip fractures from across the study sites were included, the sample was limited to patients presenting during the first wave of the pandemic, at which point testing was not applied consistently to all patients. Therefore, we performed a subgroup analysis of only the patients that tested for COVID-19 to investigate whether the trends seen in the overall population were replicated. Given possible changes to practice patterns and other confounding factors between waves, other time periods were not included. This sample had an insufficient number of COVID-19 positive patients to definitively rule out differences in postoperative outcomes, although this has been well-characterized elsewhere in the literature [9,10,11,12,13,14,15,16,17,20]. While subjects treated during the COVID-19 pandemic were matched to historical controls by age, sex, ASA, hospital, and surgical procedure, there could be other possible confounders. Importantly, frailty is a challenging factor to measure and control for, and while we did not see differences in patients’ baseline comorbidities between the study group and controls, it is possible that patients treated during the COVID-19 pandemic were more frail than historical patients, as suggested by Slulllitel and colleagues [27]. This study also focused only on operative hip fractures and, therefore, did not identify any trends in non-operative hip fracture management during the pandemic. Compared to other studies conducted during the first wave of the pandemic, the proportion of patients with positive COVID-19 tests in this study was lower, at approximately 5% [10,12,13,14,30]. This may be due to regional variation in pandemic severity.

In summary, we found no significant change in time-to-surgery, anesthesia type, 30-day mortality, or postoperative complications in patients treated for operative fragility hip fractures during the COVID-19 pandemic, compared to historical controls in New England. Shifts in discharge location may reflect the impact of COVID-19 on the healthcare system and practice patterns more broadly. Better understanding of these factors may prepare centers for the treatment of hip fractures during future public health crises, which may share characteristics with the beginning of the COVID-19 pandemic.

## Figures and Tables

**Table 1:** Baseline patient characteristics and surgery type.

Characteristic	Study Group (*n* = 64)	Controls (*n* = 64)	*p*-Value
Female sex	72% (46)	72% (46)	1.00
Age (years (SD)	81.1 ±10.9	80.9 ± 10.1	0.95
Body mass index (BMI) (kg/m^2^ ± SD) *	23.8 ±4.4	26.0 ± 4.5	0.14
White race/ethnicity ^	92% (56)	92% (58)	0.96
DNR/DNI orders	22% (14)	33% (21)	0.17
Intertrochanteric fracture	50% (32)	52% (33)	0.66
Intracapsular fracture	44% (28)	41% (26)	
Subtrochanteric fracture	5% (3)	8% (5)	
Other fracture	2% (1)	0% (0)	
Intramedullary nailing	50% (32)	50% (32)	1.00
Open reduction and internal fixation	8% (5)	8% (5)	
Total hip arthroplasty	11% (7)	11% (7)	
Hemiarthroplasty	25% (16)	25% (16)	
Percutaneous pinning	6% (4)	6% (4)	

* BMI data available for *n* = 63 patients for both groups. ^ Race/ethnicity data available for *n* = 61 in study group and *n* = 63 in control group. Reported as % (*n*) unless otherwise specified. Percentages may not add to 100% due to rounding. SD: standard deviation; DNI: do not intubate; DNR: do not resuscitate.

**Table 2:** Patient comorbidities.

Comorbidity	Study Group (*n* = 64)	Controls (*n* = 64)	*p*-Value
ASA Score	3	3	1.00
CCI (mean ± standard deviation)	6.78 ± 2.84	6.56 ± 2.52	0.65
Congestive heart failure *	30% (19)	25% (16)	0.55
Prior myocardial infarction	16% (10)	23% (15)	0.26
Hypertension	81% (52)	89% (57)	0.21
Prior stroke or transient ischemic attack	27% (17)	28% (18)	0.84
Peripheral vascular disease	17% (11)	11% (7)	0.31
Obesity	19% (12)	22% (14)	0.66
Diabetes mellitus	30% (18)	36% (23)	0.34
Asthma	19% (12)	11% (7)	0.21
Chronic obstructive pulmonary disease	20% (13)	16% (10)	0.49
Liver disease	2% (1)	6% (4)	0.17
Severe chronic kidney disease	8% (5)	3% (2)	0.24
Leukemia	0% (0)	2% (1)	0.32
Lymphoma	6% (4)	2% (1)	0.17
Solid tumor	38% (24)	31% (20)	0.46
Dementia	30% (19)	33% (21)	0.70
Osteoporosis	42% (27)	48% (31)	0.48
Connective tissue disease	11% (7)	11% (7)	1.00

* Definitions of comorbidities as per Charlson Comorbidity Index. Reported as% (*n*). ASA: American Society of Anesthesiology; CCI: Charlson Comorbidity Index.

**Table 3:** Comparison of 30-day postoperative outcomes.

Complication	Study Group (*n* = 64)	Controls (*n* = 64)	*p*-Value
Emergency department visits	19% (12)	30% (19)	0.15
Readmissions	16% (10)	17% (11)	0.81
Reoperation	2% (1)	2% (1)	1.00
Any complication	38% (24)	45% (29)	0.37
Surgical site infection	2% (1)	2% (1)	1.00
Gastrointestinal complications (vomiting)	8% (5)	5% (3)	0.47
Pneumonia	11% (7)	9% (6)	0.77
Myocardial infarction	5% (3)	3% (2)	0.65
Stroke	2% (1)	2% (1)	1.00
Sepsis	5% (3)	5% (3)	1.00
Severe bleeding	9% (6)	5% (3)	0.30
Congestive heart failure	6% (4)	6% (4)	1.00
Venous thromboembolism	3% (2)	5% (3)	0.65

## Data Availability

The data presented in this study are available on request from the corresponding author. The data are not publicly available due to patient privacy.
